# Supplementary material for: Cell death and survival due to cytotoxic exposure modelled as a two-state Ising system
Source: R Soc Open Sci. 2020 Feb 12;7(2):191578. doi: 10.1098/rsos.191578 (PMC7062046; doi:10.1098/rsos.191578)
Supplement: Supplementary information [file rsos191578supp1.docx]

SI Text

From classical Ising Hamiltonian introduced in equation (1), the partition function and the probability of finding the system in state {$s_{1}, s_{2}, \ldots, s_{N}\}$ with $s_{i}=\{0,1\}$ is obtained in a standard manner as [1]

$$Z=\sum_{s_{1}} \sum_{s_{2}} \ldots\sum_{s_{N}} e^{-\mathcal{H(}s_{1}, s_{2}, \ldots,s_{N})/k_{B}T} (S1)$$

$$P\left( s_{1}, s_{2}, \ldots,s_{N} \right)=\frac{e^{-\mathcal{H(}s_{1}, s_{2}, \ldots,s_{N})/k_{B}T}}{Z} (S2)$$

where $k_{B}$ is the Boltzmann constant and $T$ is the absolute temperature in kelvin. The corresponding average value of the order parameter (magnetization for spins or survival rate for cells) $M_{i}=\left\langle s_{i} \right\rangle$, is found from equation (1) as

$${2M}_{i}-1=k_{B}T\frac{\partial}{\partial h_{i}}\left( \ln\left( Z \right) \right) (S3)$$

This order parameter in the case of cytotoxicity corresponds to the average number of surviving cells at a given concentration of the toxic agent acting on them. In general cell survival is a function of temperature, which is typically kept constant in cell-based assays. Future experiments with temperature as a variable parameter, could further test the model. However, in cancer cell system, the most commonly used control parameter affecting cell viability is the toxic agent’s concentration. In what follows, non-interacting and interacting cell situations are discussed separately[1].

**The case of non-interacting cells:** In this case, the interaction strength, $J_{ij}$ vanishes, which corresponds to the absence of the bystander effect. The partition function in the non-interacting case $Z_{NI}$ with a constant field *h*, which represents the control parameter, is

$$Z_{NI}={(e^{-h/k_{B}T}+e^{h{/k}_{B}T})}^{N} (S4)$$

The order parameter, i.e. the survival rate for cancer cells,$M$, is calculated as

$$2M-1=\frac{k_{B}T}{N}\frac{\partial\ln\left( Z_{NI} \right)}{\partial h}=\tanh\left( \frac{h}{k_{B}T} \right) (S5)$$

From the partition function, we then calculate the probability of the system occupying each of the two states. It is especially interesting to calculate the probability of the state in which all cancerous cells are dead and there would be no correlations between cells, i.e. $s_{i}=-1$, which is obtained using equation (S4) as

$$P_{NI}\left( -1, -1, \ldots,-1 \right)=\left( \frac{e^{h{/k}_{B}T}}{e^{-h{/k}_{B}T}+e^{h{/k}_{B}T}} \right)^{N}={R(C)}^{N} (S6)$$

where $R(C)$ is the death rate as a function of drug concentration, and $S(C)=1-R(C)$ is the survival probability of a cell. Hence, magnetization and external field in the Ising model correspond to death rate, $R$, and drug concentration, $C$, respectively, and equation (S5) is written as

$$2R-1=\tanh\left( \frac{h}{k_{B}T} \right) (S7)$$

As shown below, Equation (SS7) gives very good agreement with most of the cytotoxicity assays. When $h/k_{B}T$ approaches its maximum value, all cells are dead and the death rate is at a maximum. Conversely, when $h/k_{B}T$ approaches its minimum value, all cells are in the survival state and the death rate is zero. Knowing the functional dependence of the survival rate on drug concentration, $S=S(C)$, allows one to determine the values of the model parameters for each chemotherapeutic agent and for each cancer cell line. For example, considering diffusion of drug molecules throughout the tumor with a heterogeneous microenvironment, one would expect the survival rate to follow the generalized Michaelis-Menten dynamics as

$$S\left( C \right)=\frac{1}{1+\left( \frac{C}{C_{M}} \right)^{\alpha}} (S8)$$

where $C_{M}$ is a Michaelis constant representing the drug concentration associated with reaching the half-maximal inhibition effect and the parameter $\alpha$ shows the slope of the dose-response curve and depends on the system’s heterogeneity, drug-binding efficacy and diffusion of drug molecules[2]. It can be estimated by fitting the solution to experimental data points. In cytotoxicity assays, $C_{M}$is usually denoted as IC50. Combining equations (S6) and (S8), we find that the control parameter, $h/k_{B}T$, is associated with the logarithm of the anti-cancer drug concentration according to

$$h=\frac{\alpha k_{B}T}{2}\ln\left( \frac{C}{C_{M}} \right). (S9)$$

**The case of interacting cells:** Here, the coupling constant$J_{ij}$ is non-zero, which makes the exact solution impossible to calculate analytically at least in the 3D case while it is very complicated in 2D and hence we resort to approximations. A convenient approach to solving this problem is to apply the mean-field approximation where the quadratic terms in spin fluctuations are neglected. The Hamiltonian in this case becomes

$$\mathcal{H=}\mathcal{H}_{0}+\mathcal{H}_{1} (S10)$$

where

$$\mathcal{H}_{0}=\sum_{i<j=1}^{N} J_{ij}\left\langle s_{i} \right\rangle\left\langle s_{j} \right\rangle+\sum_{i=1}^{N} h_{i} (S11)$$

$$\mathcal{H}_{1}=-\sum_{i=1}^{N} \left( 2h_{i}+J_{i}^{\mathrm{eff}} \right)s_{i} (S12)$$

and $J_{i}^{\mathrm{eff}}=\sum_{j\neq i}^{N} J_{ij}\left\langle s_{j} \right\rangle$ is the effective interaction coefficient between cells and $h_{i}$ is the direct effect of cytotoxicity on cells. From equation (S11), it follows that $\mathcal{H}_{0}$is an average value for the Hamiltonian with no effect on any particular cell directly while the first term in equation (S12) signifies the effect of the control parameter on the $i$th cell $s_{i}$ and the second term can be interpreted as the average bystander effect from all other cells on the $i$th cell $s_{i}$[1,3]. Hence, the partition function $Z$ given by equation (S1) becomes

$$Z=\prod_{i=1}^{N} \left( 1+e^{\left( 2h_{i}+J_{i}^{\mathrm{eff}} \right)/k_{B}T} \right) (S13)$$

Note that equation (S10) is a general result valid for any drug concentration distribution used in numerical computations[1]. To derive some analytical results, however, we need to assume again a uniform drug distribution *via* $h=h_{i}$ , which leads to the uniform magnetization,$M=M_{i}$, representing an average survival response of the cell culture, and the effective constant interaction strength, *J*. As a result, the partition function and the order parameter are found after several steps of calculations as

$$Z=\left[ 2e^{-\lambda M(M-1)}\cosh\left( \frac{h}{k_{B}T}+\lambda M \right) \right]^{N} (S14)$$

and

$$2M-1=\tanh\left( \frac{h}{k_{B}T}+\lambda M \right) (S15)$$

where $\lambda=J/2k_{B}T$.

**Ising model:** The Ising model, first introduced to statistical physics by Wilhelm Lenz in 1920, is one of the simplest examples of dynamical systems undergoing a phase transition [4]. An Ising system must be at least two-dimensional in space, in the absence of an external magnetic field, for a spontaneous phase transition to occur, i.e., no phase transition takes place in one-dimensional Ising systems[5–13]. The Ising model is a standard mathematical model of a phase transition in a lattice of spins ½ (with only two states: +1/2 and -1/2) where each spin is allowed to interact with its neighbors[14,15]. Below a characteristic temperature called the Curie temperature, the Ising system exhibits a ferromagnetic phase (spins are aligned along the same axis) while above this temperature a paramagnetic phase is stable where spins are disordered and no net magnetization of the sample exists. The transition from a non-magnetized state to a magnetized state depends on both temperature and the applied magnetic field’s strength. Generally, the Ising model corresponds to any N-dimensional lattice whose each site is occupied by a spin with two possible states, pointing either ‘up’ or ‘down’[16]. The mathematical notation used for the spin variable is $s_{i}=\pm1/2$ where +1/2 refers to spin up and -1/2 refers to spin down. In this study, the Ising model has been applied to both interacting and non-interacting generalized spin systems[14–21]. We arbitrarily assign a spin-up state to a live cell while a spin-down state to a dead cell. This bystander effect[1,22–26] may account for the cell-cell interactions in a manner similar to spin-spin interactions between neighboring spins, hence we propose a model whereby a damaged cancer cell might affect the survival status of the neighboring cells. Due to the influence of neighboring cells on individual cell fate, the drug concentration reduces the chance of survival of other cancer cells in the neighborhood[1]. These types of interactions are distance-dependent, so the farther apart the neighboring cells are, the weaker their intercellular interactions[1,22–26].

Landau Theory of Phase Transitions: Landau theory of phase transitions is commonly used to study phase transitions in magnetic materials. Unlike the Ising model, Landau theory doesn’t directly and explicitly rely on the interactions between individual spins. Instead, it approximates their interactions by introducing a single average effect. These systems are described by a free energy function, $F(M)$, a power series of the magnetization, $M$, as an order parameter. To guarantee equal spin orientation in the absence of the external magnetic field, the even powers in the summation are only allowed, so that the free energy of Landau theory would read as

$$F\left( M,T \right)=F_{0}-hM+\frac{A}{2}M^{2}+\frac{B}{4}M^{4}\mathcal{+O}\left( M^{6} \right) (S16)$$

where $A=a\left( T-T_{C} \right)$ and $h$ is the external magnetic field as a control parameter, $T$ is temperature and $T_{C}$ is the critical temperature for this system that marks the phase transition point[22–24,27]. Since the free energy is expected to be at a minimum at thermodynamic equilibrium, three different situations should be considered. The first one is when $A<0$, in which the free energy will have two minima. The second case is when $A>0$, hence the free energy is characterized by only one minimum taking place at the origin ($M=0$), and finally the third case when $A=0$ which will be a transition between the first and second case. Figure S1 depicts all the three conditions of the Landau free energy in the absence of an external field[22–24,27]. Solving the Landau model for equilibrium conditions gives the following results for magnetization, external field and the susceptibility,$\chi$, near critical temperature

$$\frac{\partial F(M)}{\partial M}=0 \mathrm{and} h=0, M=\left\{ {\pm\left( \frac{a(T_{c}-T)}{B} \right)^{1/2} T<T_{c} \atop0 T>T_{c}} \right., \beta=\frac{1}{2}$$

$$\frac{\partial F(M)}{\partial M}=0 \left( Close to T_{c} \right), h=BM^{3}, \delta=3 (S17)$$

$$\chi^{-1}=\frac{\partial^{2}F}{\partial M^{2}}>0, \chi^{-1}=\left\{ \begin{aligned} a({T-T}_{c}) T>T_{c} \\ \\ -2a(T_{c}-T) T<T_{c} \end{aligned} \right., \gamma=1$$

So, close to $T_{c}$ magnetic field and the magnetization follow $h\approx M^{3} .$in our model, $T_{c}$ can be corresponded to IC50 value and we showed in figure S1a that the model is working close to $T_{c}$ and Landau theory of phase transition and Ising model for cytotoxicity are correlating.

Experimental Data: All these 13 cytotoxic drugs have a defined principal molecular target; they belong to a specific cytotoxic classification and they are used against a particular cancer type in clinical trial experimental assays[28–32]. In addition, information about all the 66 cell lines has been obtained from the American Type Culture Collection (ATCC). The cell lines were cultured in ATCC-recommended media. Using the method of ATPlite 1step, the cell proliferation assays were extracted[33,34]. The exposure time was 72 hours for all compounds except for Vincristine and Tazemetostat where it was 120 hours. After 72 hours, inhibition of growth in the presence of these compounds was determined. The experiments were also carried out for the same cell lines without adding any compound but only by adding vehicle (DMSO) to the cells in order to provide controls for comparison. Between the two untreated profiles, growth inhibition is given for an increasing concentration of drug every 72 hours. Each experimental assay was repeated twice while increasing the concentration and the data between two untreated cells were measured four times. For most of the compounds the following quantities are reported: IC50 (note that the IC50 is the same as $C_{M}$in equation (5), i.e. the inhibition concentration where the response is reduced by half; GI50, the growth inhibition that denotes the drug concentration at which it causes 50% reduction in cancer cells growth; and LD50, the lethal dose that represents the amount of drug which kills 50% of a test sample. The maximum concentration tested for the compounds was 31.6 $\mu M$ and no further increases in the concentration were made[33,34].

Data pre-processing: In the Table. S1 the experimental IC50 values of the studied cell lines, collecting by the collaborators in the Netherlands Translational Research Center B.V. (Oncolines), are listed[33,34]. The empty cells shows that the IC50 was higher than 31.6 $\mu M$ and they were not measured experimentally. For analysing the data, the death rate is obtained using the cell survival rates, $R(C)=1-S(C)$. Using the cell response-Ising model, the death rates has been fitted with the equation (7). For obtaining the fitting parameters in the interacting and non-interacting cells, Python and MATLAB software have been used and the codes can be found in the Dryad repository [35].

**Cell lines agreement to the Ising model:** In Table S3, the studied cell lines are listed for $13$ drugs in an increasing order of the correlation coefficient with the Ising cytotoxicity model. Cell lines having good agreement with the model are shown in green, filtering by $R^{2}>0.5$ and the susceptibility $\chi<10$, while cell lines with poor agreement are shown in red labeled by a cross mark.

**Interaction parameter,** $\boldsymbol{\lambda}$**:** Figure S3 compares the fitting parameter averaged over all 66 cell lines, $a, b, c, d$ and $\lambda$ for each cytotoxic drug in two cases of interacting and non-interacting cells, respectively, followed by the average and error bars. It can be seen that the two cases are fairly consistent in the values of a, b, c, and d, and the important interaction parameter, $\lambda$, changes between $2.1$ to $6.1$ on average. In fact, since $\lambda$ is greater than $\lambda_{c}$, the cell-cell interactions are not significant.

SI Appendix

| a)    b)   |
| --- |

Figure S1.(a) Plot of magnetization as a function of external field when ($q=0)$ for different temperature limits $T<T_{C}, \left( k_{B}T=0.1 \right)$, $T=T_{C}\left( k_{B}T=1 \right)$and $T>T_{C}, \left( k_{B}T=1.9 \right)$. (b) Free energy of Landau theory for different conditions in temperature, ($h=0$).

Figure S2. Fitting parameters for equation (7), a, b, c, d and $R^{2}$ for the cytotoxic drugs tested. Error bars show the standard deviation error.

| .  |
| --- |

**Figure S3.** Ising cytotoxic model best-fit parameters for the interacting (in black) and non-interacting (in red) cases of cancer cells exposed to chemotherapy drugs.

Table S1.The anti-cancer compounds used in experimental assays[28–32].

| **Name** | **Main target and Classification of cytostatic** | **Clinical trial use** |
| --- | --- | --- |
| Busulfan | DNA alkylating  (Alkylating agents) | bone marrow transplantation, especially in chronic myelogenous leukemia (CML) and other leukemias, lymphomas, and myeloproliferative disorders |
| Methotrexate | folate synthesis  (Antimetabolites) | breast cancer, leukemia, lymphoma, lung cancer, and osteosarcoma |
| Paclitaxel | Tubulin  (Anti-microtubule agents-Taxanes) | ovarian cancer, breast cancer, Kaposi sarcoma, lung cancer, cervical cancer, and pancreatic cancer |
| Vincristine | Tubulin (Anti-microtubule agents-Vinca alkaloid) | acute lymphocytic leukemia, acute myeloid leukemia, Hodgkin's disease, neuroblastoma, and small cell lung cancer |
| Doxorubicin | topoisomerase II (Antitumor antibiotics) | breast cancer, bladder cancer, Kaposi's sarcoma, lymphoma, and acute lymphocytic leukemia |
| Cisplatin | DNA damage  (Others/platin-like) | testicular cancer, ovarian cancer, breast cancer, bladder cancer, cervical cancer, head and neck cancer, esophageal cancer, lung cancer, mesothelioma, brain tumors and neuroblastoma |
| Irinotecan | topoisomerase I  (Topoisomerase inhibitors) | Treat colon cancer and small cell lung cancer |
| Bortezomib | proteasome | multiple myeloma and mantle cell lymphoma |
| Tazemetostat | EZH2 | lymphoma (non-Hodgkin lymphoma adult patients with certain genetically defined solid tumors, including INI1-negative tumors and synovial sarcoma, and patients with mesothelioma characterized by BAP loss of function) |
| **Specific kinase inhibitors** | | |
| Afatinib | EGFR | [non-small cell lung carcinoma (NSCLC)](https://en.wikipedia.org/wiki/Non-small_cell_lung_carcinoma) with common epidermal growth factor receptor (EGFR) mutation |
| Idelalisib | PI3K | [hematological malignancies](https://en.wikipedia.org/wiki/Tumors_of_the_hematopoietic_and_lymphoid_tissues) |
| Palbociclib | CDK 4/6 | ER-positive and HER2-negative breast cancer developed |
| Trametinib | MEK | advanced malignant melanoma |

Table S2. Excluded cell lines due to $R^{2}<0.5$ and the susceptibility $\chi>10$.

| **Cell line** | **Bortezomib** | **Vincristine** | **Doxorubicin** | **Methotrexate** | **Paclitaxel** | **Irinotecan** | **Cisplatin** | **Afatinib** | **Tremetinib** | **Idelalisib** | **Tazemetostat** | **Palbociclib** | **Busulfan** | **Cell line** | **Bortezomib** | **Vincristine** | **Doxorubicin** | **Methotrexate** | **Paclitaxel** | **Irinotecan** | **Cisplatin** | **Afatinib** | **Tremetinib** | **Idelalisib** | **Tazemetostat** | **Palbociclib** | **Busulfan** |
| --- | --- | --- | --- | --- | --- | --- | --- | --- | --- | --- | --- | --- | --- | --- | --- | --- | --- | --- | --- | --- | --- | --- | --- | --- | --- | --- | --- |
| **769-P** |  |  |  |  |  |  |  |  |  |  | X |  |  | **MCF7** |  |  |  |  |  |  |  |  |  |  |  | X |  |
| **786-O** |  |  |  |  |  |  |  |  | X | X | X |  |  | **C-33 A** |  |  |  |  |  |  |  | X |  | X | X |  | X |
| **A-498** |  |  |  |  |  |  |  |  |  |  | X |  |  | **DoTc2 4510** |  |  |  |  |  |  |  | X |  | X | X |  | X |
| **A-704** |  |  |  |  |  |  |  |  | X |  |  |  | X | **CAL 27** |  |  |  |  |  |  |  |  |  |  |  |  | X |
| **ACHN** |  |  |  |  |  |  |  | X |  |  | X |  |  | **COLO 205** |  |  |  |  |  |  |  |  |  |  | X |  | X |
| **A-172** |  |  |  |  |  |  |  |  |  | X |  |  | X | **DLD-1** |  |  |  |  |  |  |  |  | X |  |  |  |  |
| **CCRF-CEM** |  |  |  |  |  |  |  |  |  |  | X |  |  | **HCT 116** |  |  |  |  |  |  |  |  |  |  |  |  | X |
| **Jurkat E6.1** |  |  |  |  |  |  |  |  | X |  |  |  |  | **HCT-15** |  |  |  |  |  |  |  |  |  |  | X |  | X |
| **KU812** |  |  |  |  |  |  |  |  |  | X |  | X |  | **LS 174T** |  |  |  |  |  |  |  |  |  |  |  |  |  |
| **SUP-T1** |  |  |  |  |  |  |  |  | X |  | X | X | X | **LoVo** |  |  |  |  |  |  |  |  |  | X | X |  |  |
| **SR** |  |  |  |  |  |  |  |  |  | X |  |  |  | **RKO** |  |  |  |  |  |  |  |  |  |  |  |  | X |
| **MOLT-4** |  |  |  |  |  |  |  |  |  |  |  |  |  | **SW48** |  |  |  |  |  |  |  |  |  |  |  | X |  |
| **K-562** |  |  |  |  |  |  |  |  |  | X | X |  |  | **SW480** |  |  |  |  |  |  |  |  |  | X |  |  | X |
| **A-204** |  |  |  |  |  |  | X |  | X |  | X | X |  | **SW620** |  |  |  |  |  |  |  |  |  |  |  | X |  |
| **SJCRH30** |  |  |  |  |  |  |  |  |  |  | X |  | X | **SW948** |  |  |  |  |  |  | X |  |  |  | X |  | X |
| **A375** |  |  |  |  |  |  |  |  |  | X | X |  |  | **SNU-C2B** |  |  |  |  |  |  |  | X |  | X |  |  | X |
| **COLO 829** |  |  |  |  |  |  |  |  |  |  | X | X | X | **T24** |  |  |  |  |  |  |  |  |  | X | X | X |  |
| **MeWo** |  |  |  |  |  |  | X |  |  |  |  |  |  | **RT4** |  |  |  |  |  |  |  |  |  |  |  | X |  |
| **RPMI-7951** |  |  |  |  |  |  |  |  |  |  |  | X | X | **J82** |  |  |  |  |  |  |  |  |  |  |  | X | X |
| **A388** |  |  |  |  |  |  |  | X |  |  |  |  | X | **Daoy** |  |  |  |  |  |  |  |  | X |  |  |  |  |
| **A-427** |  |  |  |  |  |  |  |  |  |  |  | X |  | **U-87 MG** |  |  |  |  |  |  |  |  | X |  | X | X | X |
| **A-549** |  |  |  |  |  |  |  |  |  |  | X |  |  | **T98G** |  |  |  |  |  |  |  |  |  |  |  | X | X |
| **NCI-H460** |  |  |  |  |  |  |  |  |  |  |  |  |  | **SK-N-AS** |  |  |  |  |  |  |  |  |  |  | X | X |  |
| **SHP-77** |  |  | X |  |  | X |  |  | X | X |  |  | X | **SK-N-FI** |  |  | X |  |  |  |  |  |  |  |  | X |  |
| **NCI-H82** |  |  |  |  |  |  |  |  | X | X |  |  | X | **MG-63** |  |  |  |  |  |  |  |  |  |  |  | X | X |
| **AN3 CA** |  |  |  |  |  | X |  |  |  | X |  | X |  | **U-2 OS** |  |  |  |  |  |  |  |  | X | X |  |  | X |
| **AsPC-1** |  |  |  |  |  |  |  | X |  |  |  | X | X | **VA-ES-BJ** |  |  |  |  |  |  |  |  |  |  |  | X |  |
| **BxPC-3** |  |  |  |  |  |  |  |  | X |  |  | X | X | **DU 145** |  |  |  |  |  |  |  | X | X |  |  |  |  |
| **MIA PaCa-2** |  |  |  |  |  |  |  |  |  |  |  |  |  | **LNCaP FGC** |  |  |  |  |  |  |  |  | X |  |  | X | X |
| **AU-565** |  |  |  |  |  |  |  |  | X |  | X |  |  | **TT** |  |  |  |  |  |  | X |  |  |  |  | X | X |
| **BT-20** |  |  |  |  |  |  |  |  |  | X | X | X | X | **FaDu** |  |  |  |  |  |  |  | X |  | X |  | X | X |
| **BT-549** |  |  |  |  |  |  |  |  | X |  | X | X |  | **OVCAR-3** |  |  |  |  |  |  |  |  |  |  |  | X |  |
| **Hs 578T** |  |  |  |  |  | X |  |  | X |  |  |  |  | **PA-1** |  |  |  |  |  |  |  |  | X | X | X |  | X |

**SI References:**

1. Vassiliev ON. 2014 A model of the radiation-induced bystander effect based on an analogy with ferromagnets. Application to modelling tissue response in a uniform field. *Physica A* **416**, 242–251. (doi:10.1016/j.physa.2014.08.052)

2. Sun X, Bao J, Shao Y. 2016 Mathematical Modeling of Therapy-induced Cancer Drug Resistance: Connecting Cancer Mechanisms to Population Survival Rates. *Sci Rep* **6**, 22498. (doi:10.1038/srep22498)

3. Powathil GG, Munro AJ, Chaplain MA, Swat M. 2016 Bystander effects and their implications for clinical radiation therapy: Insights from multiscale in silico experiments. *J. Theor. Biol.* **401**, 1–14. (doi:https://doi.org/10.1016/j.jtbi.2016.04.010)

4. Brush SG. 1967 History of the Lenz-Ising Model. *Rev. Mod. Phys.* **39**, 883–893. (doi:10.1103/RevModPhys.39.883)

5. Davies PC, Demetrius L, Tuszynski JA. 2011 Cancer as a dynamical phase transition. *Theor Biol Med Model* **8**, 30. (doi:10.1186/1742-4682-8-30)

6. Weinberg RA. 2013 *The Biology of Cancer, 2nd Edition*. 2nd edition. New York: W.W. Norton & Company.

7. Smith A-S. 2010 Physics challenged by cells. *Nature Phys* **6**, 726–729. (doi:10.1038/nphys1798)

8. Alberts B, Johnson A, Lewis J, Raff M, Roberts K, Walter P. 2002 *Molecular Biology of the Cell*. 4th edn. Garland Science.

9. Ising T, Folk R, Kenna R, Berche B, Holovatch Y. 2017 The Fate of Ernst Ising and the Fate of his Model. *ArXive* **21**.

10. Kipnis AYa, Yavelov BE, Rowlinson JS. 1996 *Van Der Waals and Molecular Science*. United Kingdom: Oxford University Press.

11. Onsager L. 1944 Crystal Statistics. I. A Two-Dimensional Model with an Order-Disorder Transition. *Phys. Rev.* **65**, 117–149. (doi:10.1103/PhysRev.65.117)

12. Kramers HA, Wannier GH. 1941 Statistics of the Two-Dimensional Ferromagnet. Part II. *Phys. Rev.* **60**, 263–276. (doi:10.1103/PhysRev.60.263)

13. Heisenberg W. 1928 Zur Theorie des Ferromagnetismus. *Zeitschrift fur Physik* **49**, 619–636. (doi:10.1007/BF01328601)

14. Selinger J. 2016 *Introduction to the Theory of Soft Matter: From Ideal Gases to Liquid Crystals*. 1st edn. Springer International Publishing.

15. Torquato S. 2011 Toward an Ising Model of Cancer and Beyond. *Phys Biol* **8**, 015017. (doi:10.1088/1478-3975/8/1/015017)

16. Andreassen CN, Alsner J. 2009 Genetic variants and normal tissue toxicity after radiotherapy: A systematic review. *Radiother Oncol.* **92**, 299–309. (doi:10.1016/j.radonc.2009.06.015)

17. Yeomans JM. 1991 *Statistical mechanics of phase transitions*. Clarendon Press.

18. Goldenfeld N. 1992 *Lectures on phase transitions and the renormalization group*. 1st edn. Westview Press.

19. Ma S-K. 1985 *Statistical Mechanics*. World Scientic Publishing Co Inc.

20. Katzgraber HG. 2009 Introduction to Monte Carlo Methods. *arXiv:0905.1629 [cond-mat, physics.]*

21. Salinas SRA. 2001 *Introduction to statistical physics*. New York : Springer.

22. Landau LD. 1937 On the theory of phase transitions. *Zh.Eksp.Teor.Fiz.* **7**, 19–32. (doi:doi:10.1038/138840a0)

23. Tolédano J-C, Tolédano P. 1987 *The Landau Theory of Phase Transitions: Application to Structural, Incommensurate, Magnetic and Liquid Crystal Systems*. (doi:10.1142/0215)

24. Cowley RA. 1980 Structural phase transitions I. Landau theory. *Advances in Physics* **29**, 1–110. (doi:10.1080/00018738000101346)

25. Zimmerman JR, Foster MR. 1957 Standardization of N.M.R. High Resolution Spectra. *J. Phys. Chem.* **61**, 282–289. (doi:10.1021/j150549a006)

26. Yang CN. 1952 The Spontaneous Magnetization of a Two-Dimensional Ising Model. *Phys. Rev.* **85**, 808–816. (doi:https://doi.org/10.1103/PhysRev.85.808)

27. Lifshitz EM. 2013 *Statistical Physics : Theory of the Condensed State.* San Diego : Butterworth-Heinemann.

28. Wishart DS *et al.* 2018 DrugBank 5.0: a major update to the DrugBank database for 2018. *Nucleic Acids Res.* **46**, D1074–D1082. (doi:10.1093/nar/gkx1037)

29. Law V *et al.* 2014 DrugBank 4.0: shedding new light on drug metabolism. *Nucleic Acids Res* **42**, D1091–D1097. (doi:10.1093/nar/gkt1068)

30. Knox C *et al.* 2011 DrugBank 3.0: a comprehensive resource for ‘Omics’ research on drugs. *Nucleic Acids Res* **39**, D1035–D1041. (doi:10.1093/nar/gkq1126)

31. Wishart DS, Knox C, Guo AC, Cheng D, Shrivastava S, Tzur D, Gautam B, Hassanali M. 2008 DrugBank: a knowledgebase for drugs, drug actions and drug targets. *Nucleic Acids Res.* **36**, D901-906. (doi:10.1093/nar/gkm958)

32. Wishart DS, Knox C, Guo AC, Shrivastava S, Hassanali M, Stothard P, Chang Z, Woolsey J. 2006 DrugBank: a comprehensive resource for in silico drug discovery and exploration. *Nucleic Acids Res.* **34**, D668-672. (doi:10.1093/nar/gkj067)

33. Uitdehaag JCM *et al.* 2016 Cell Panel Profiling Reveals Conserved Therapeutic Clusters and Differentiates the Mechanism of Action of Different PI3K/mTOR, Aurora Kinase and EZH2 Inhibitors. *Mol. Cancer Ther.* **15**, 3097–3109. (doi:10.1158/1535-7163.MCT-16-0403)

34. Uitdehaag JCM *et al.* 2014 Comparison of the cancer gene targeting and biochemical selectivities of all targeted kinase inhibitors approved for clinical use. *PLoS ONE* **9**, e92146. (doi:10.1371/journal.pone.0092146)

35. Arbabi Moghadam S, Rezania V, Tuszynski S. 2019 Cell Death and Survival Due to Cytotoxic Exposure Modeled as a Two-State Ising System. , Dryad Digital Repository. (doi:https://doi.org/10.5061/dryad.4qrfj6q6d)
